# (−)-Epigallocatechin-3-Gallate Diminishes Intra-and Extracellular Amyloid-Induced Cytotoxic Effects on Cholinergic-like Neurons from Familial Alzheimer’s Disease PSEN1 E280A

**DOI:** 10.3390/biom11121845

**Published:** 2021-12-08

**Authors:** Viviana Soto-Mercado, Miguel Mendivil-Perez, Carlos Velez-Pardo, Marlene Jimenez-Del-Rio

**Affiliations:** Neuroscience Research Group, Medical Research Institute, Faculty of Medicine, University of Antioquia (UdeA), Calle 70 No. 52-21, and Calle 62 # 52-59, Building 1, Room 412, SIU, Medellin 050010, Colombia; viviana.soto@udea.edu.co (V.S.-M.); miguel.mendivil@udea.edu.co (M.M.-P.); calberto.velez@udea.edu.co (C.V.-P.)

**Keywords:** cholinergic neurons, epigallocatechin-3-gallate, phytochemical, polyphenol, familial Alzheimer disease, PSEN1, E280A mutation, sAPPβf, TAU, oxidative stress, apoptosis, neuronal dysfunction

## Abstract

Alzheimer’s disease (AD) is a complex neurodegenerative disease characterized by functional disruption, death of cholinergic neurons (ChNs) because of intracellular and extracellular Aβ aggregates, and hyperphosphorylation of protein TAU (p-TAU). To date, there are no efficient therapies against AD. Therefore, new therapies for its treatment are in need. The goal of this investigation was to evaluate the effect of the polyphenol epigallocatechin-3-gallate (EGCG) on cholinergic-like neurons (ChLNs) bearing the mutation E280A in PRESENILIN 1 (PSEN1 E280A). To this aim, wild-type (WT) and PSEN1 E280A ChLNs were exposed to EGCG (5–50 μM) for 4 days. Untreated or treated neurons were assessed for biochemical and functional analysis. We found that EGCG (50 μM) significantly inhibited the aggregation of (i)sAPPβf, blocked p-TAU, increased ∆Ψm, decreased oxidation of DJ-1 at residue Cys106-SH, and inhibited the activation of transcription factor c-JUN and P53, PUMA, and CASPASE-3 in mutant ChLNs compared to WT. Although EGCG did not reduce (e)Aβ42, the polyphenol reversed Ca^2+^ influx dysregulation as a response to acetylcholine (ACh) stimuli in PSEN1 E280A ChLNs, inhibited the activation of transcription factor NF-κB, and reduced the secretion of pro-inflammatory IL-6 in wild-type astrocyte-like cells (ALCs) when exposed to mutant ChLNs culture supernatant. Taken together, our findings suggest that the EGCG might be a promising therapeutic approach for the treatment of FAD.

## 1. Introduction

Alzheimer’s disease (AD) is a multifaceted progressive neurodegenerative disorder [1] characterized by loss of memory and cognitive impairment caused by the deterioration of the cholinergic system, accumulation of insoluble intracellular (iAβ) and extracellular forms of amyloid β (eAβ, mostly eAβ42), intracellular aggregation of the microtubule protein TAU in neurofibrillary tangles, as well as neuronal cell death, synaptic dysfunction, and neuroinflammation [2,3,4]. The Aβ42 peptide originates from the sequential cleavage of the amyloid precursor protein (APP) by the beta-site amyloid precursor protein cleaving enzyme 1 (BACE1), and by the gamma-site aspartyl protease γ-secretase [5]. This last protein is a membrane-embedded protease complex composed of presenilins (PSEN1 and PSEN2), presenilin enhancer 2 (PEN2), anterior pharynx-defective 1 (APH-1), and nicastrin subunits, with presenilins as the catalytic component containing two transmembrane aspartates in the active site [6]. While there are no known mutations in *PEN2*, *APH-1*, and *NICASTRIN*, more than 300 variants have been reported in the *PSEN 1* gene (https://www.alzforum.org/mutations/psen-1; accessed on 15 October 2021) resulting mostly in the overproduction of eAβ42 [7]. Unfortunately, the Glu280Ala (p. E280A, c. 839A > C, exon 8) mutation in PSEN1 has been reported as causal of familial AD (FAD) with complete penetrance in large kindred localized in Antioquia, Colombia [8,9,10]. Despite prevention efforts and treatment approaches [11,12], and advanced knowledge of the neuropathology of PSEN1 E280A (e.g., [13,14]), there are no efficient therapies to date. Therefore, new therapies for the treatment of FAD patients are in need. 

Recently, we have recreated the molecular pathogenesis of FAD PSEN1 E280A mutation in vitro [15]. Umbilical cord mesenchymal stem cells-derived PSEN1 E280A Cholinergic-like neurons (PSEN1 E280A ChLNs) exhibit early intracellular accumulation of soluble APPβ fragments (iAPPβf, but not Aβ42 peptide), oxidized DJ-1 (at residue Cys^106^SO_3_) indicative of oxidative stress (OS), and hyperphosphorylation of protein TAU [15]. Also, PSEN1 E280A ChLNs display loss of the mitochondrial membrane potential (∆Ψm), activation of apoptogenic proteins, and DNA fragmentation, all markers of apoptosis—a type of regulated cell death. Moreover, mutant ChLNs secrete eAβ42 and displayed Ca^2+^ flux dysregulation when challenged to acetylcholine (ACh) compared to wild-type (WT) ChLNs [15]. Therefore, PSEN1 E280A ChLNs provide an excellent model for screening candidate molecule(s)/drug(s).

In response to the urgent need for available drugs for the treatment of AD [16], we used PSEN1 E280A ChLNs as a cellular system to evaluate potential natural (phytochemicals) or synthetic compounds to advance neuroprotective therapies (e.g., [17]). Lastly, natural products have been postulated to reduce the accumulation and toxic effects of Aβ peptides via antioxidant activity, secretase- or structure-dependent pathways, metal chelation, and preventing Aβ aggregation [18,19,20]. Specifically, the green tea polyphenol epigallocatechin-3-gallate (EGCG)—a subclass of flavan-3-ols has shown anti-amyloidogenic, antioxidant, anti-BACE1 secretase, metal chelation, neuroprotective and anti-inflammatory activity in vivo and in vitro [19,20,21,22,23,24,25], among other functions. Despite these observations, no data are available to establish whether EGCG can reverse the neuropathological markers in the aforementioned PSEN1 E280A ChLNs model [15]. Moreover, it is not yet known whether EGCG might be able to block aggregation of iAPPβf, oxidation of DJ-1, phosphorylation of protein TAU, neuronal apoptosis, decrease toxic effect of eAβ42, and/or prevent Ca^2+^ dysregulation in those mutant cholinergic neurons. 

Neuroinflammation plays an important role in AD [4] resulting from the hyperactivation of microglia and astrocytes that release pro-inflammatory cytokines due to the neurological insults caused by Aβ plaques, leading to synaptic dysfunction and neuronal death [26]. Due to the importance of astrocytes in brain homeostasis and neuroinflammation, they have become the focus of active research in AD [27,28]. Recently, we have shown that functional astrocyte-like cells (ALCs, ~59% GFAP+/S100β+ responsive to glutamate-induced Ca^2+^ inward stimuli) can be derived from menstrual stromal cells (MenSCs) by direct transdifferentiation method using commercial Gibco^®^ Astrocyte medium for 7 days [29]. Interestingly, it has been shown that Aβ42 can trigger reactive astrogliosis, releasing neuroinflammatory cytokines (e.g., IL-6) and via activation of transcription factor NF-κB [30]. However, it is not yet known whether EGCG diminishes the reactive astrogliosis induced by eAβ42 in ALCs. 

To get insight into these issues, we have selected the commercially available (−)-epigallocatechin-3-gallate (hereafter EGCG) to evaluate its effect on wild-type and PSEN1 E280A ChLNs concerning the isAPPβf accumulation, TAU phosphorylation, OS, cell death, and Ca^2+^ neuronal dysfunction in ChLNs. In addition, we also want to assess whether EGCG reduces wild-type reactive astrocytes induced by PSEN1 E280A ChLNs culture supernatant. We demonstrate for the first time that EGCG can reverse PSEN1 e280A cholinergic dysfunction not only by preventing structural and functional damage of neurons, and OS-induced cell death signaling induced by (i)APPβf/eAβ42 but also by acting as an anti-inflammatory agent. These findings might favor the use of EGCG as a potential lead compound candidate for FAD therapy. 

## 2. Materials and Methods

### 2.1. Cholinergic-like Neuron (ChLN) Differentiation

ChLN differentiation was performed according to ref. [31]. The WT (TBC# WJMSC-11) and PSEN1 E280A (TBC# WJMSC-12) [15] MSCs were seeded at 1–1.5 × 10^4^ cells/cm^2^ in laminin-treated culture plates for 24 h in regular culture medium (RCm). The medium was removed, and cells were incubated in cholinergic differentiation medium (*Cholinergic-N-Run* medium, hereafter Ch-N-Rm) containing DMEM/F-12 media 1:1 Nutrient Mixture (Gibco cat# 10565018; 1204 N Western St, Suite C, Amarillo, TX, USA), 10 ng/mL basic fibroblast growth factor (bFGF) recombinant human protein (Gibco Cat# 13256029), 50 µg/mL sodium heparin (Hep, Sigma-Aldrich cat# H3393; 3050 Spruce Street, St. Louis, MO 63103, USA), 0.5 µM all-trans retinoic acid, 50 ng/mL sonic hedgehog peptide (SHH, Sigma cat# SRP3156) and 1% FBS at 37 °C for 7 days. After this process of transdifferentiation, the cells were labeled as WT PSEN1 or PSEN1 E280A ChLNs. Since Ch-N-Rm contains several factors that might interfere with the experiment interpretation and measurements, WT PSEN1 and PSEN1 E280A ChLNs (obtained after 7 days in Ch-N-Rm) were left in a regular culture medium (RCm) for 4 additional days of post transdifferentiation.

### 2.2. Astrocyte-like Cells (ALCs) Differentiation

Isolation of mesenchymal stromal cells (MenSCs) derived from wild-type human menstrual blood were obtained from tissue bank code (TBC# 69308)-UdeA [29]. For astrocyte differentiation, 1 × 10^4^ WT MenSCs/cm^2^ were seeded in 25 cm^2^ culture flasks in regular culture medium (RCm, DMEM low glucose Sigma cat# D6046 media supplemented with 10% FBS) until reach 40% of confluence. Then, the medium was replaced, and cells were incubated either in DMEM low glucose media supplemented with 2% FBS (minimal culture medium, thereafter MCm) or Astrocyte medium^®^ (GIBCO^®^, cat#A1261301, 1204 N Western St., Suite C, Amarillo, TX, USA) for 7 days.

### 2.3. Assay Protocol

The methodology for both WT and PSEN1 E280A ChLNs cell culture assays was the same. Initial EGCG screening was performed at least twice in triplicate between 5 μM–50 μM. Subsequently, EGCG (50 μM) was established as an optimal concentration for further experiments. ChLNs were divided into two groups: (i) untreated; (ii) treated with EGCG (50 μM). 

### 2.4. Immunofluorescence Analysis

The analysis of Alzheimer’s disease-, oxidative stress- and cell death-related markers, was exactly performed as described elsewhere [15]. Briefly, the cells treated under different conditions were fixed with 4% paraformaldehyde for 20 min, followed by Triton X-100 (0.1%) permeabilization and 10% bovine serum albumin (BSA) blockage. Cells were incubated overnight with primary antibodies against APP751 and/or protein amyloid β1-42 (1:500; clone 6E10 cat# 803014, Biolegend, 9727 Pacific Heights Blvd, San Diego, CA 92121, USA), total TAU (1: 500; t-Tau; cat# T6402, Sigma-Aldrich, 3050 Spruce Street, St. Louis, MO 63103, USA), and phospho-TAU (p-Tau, 1:500, Ser202/Thr205, cat# MN1020 (AT8), Thermo Fisher Scientific, 168 Third Avenue, Waltham, MA, USA); and primary antibodies against oxidized DJ-1 (1:500; ox(Cys106)DJ-1; spanning residue C106 of human PARK7/DJ1; oxidized to produce cysteine sulfonic (SO3) acid; cat # ab169520, Abcam, Discovery Drive Cambridge Biomedical Campus Cambridge CB2 0AX, UK). To assess cell death, we used primary antibodies against p53-upregulated modulator of apoptosis (1:500; PUMA, cat# ab-9643, Abcam), p53 (1:500; cat# MA5-12453, Millipore, 3050 Spruce Street, St. Louis, Mo, 63304, USA), phospho-c-Jun (1:250; c-Jun (S63/73) cat# sc-16312, Santa Cruz Biotechnology, 2145 Delaware Avenue, Santa Cruz, CA 95060, USA), and caspase-3 (1:250; cat # AB3623, Millipore). To evaluate the reactivation of astrocytes, we used primary antibodies against NF-κB (rabbit anti-NF-κB/p65, Thermo cat# PA5-16545). After exhaustive rinsing, we incubated the cells with secondary fluorescent antibodies (DyLight 488 and 594 horse anti-rabbit, -goat and -mouse, cat DI 1094, DI 3088, and DI 2488, respectively) at 1:500. The nuclei were stained with 1 µM Hoechst 33,342 (Life Technologies, 5791 Van Allen Way, Carlsbad, CA 92008, USA), and images were acquired on a Floyd Cells Imaging Station microscope (catalogue 4471136, Thermo Fisher Scientific, 168 Third Avenue, Waltham, MA, USA).

### 2.5. Flow Cytometry Analysis 

After each treatment, cells were detached using trypsin and centrifuged for 10 min at 2000 rpm. Then, cells were washed with PBS and permeabilized with 0.2% Triton X-100 plus 1.5% bovine serum albumin (BSA) for 30 min. After, cells were incubated with primary antibodies against APP751 and/or protein amyloid β1-42 (clone 6E10, 1:200), phospho-TAU (AT8; 1:200), oxidized DJ-1 (1:200), PUMA (1:200), p53 (1:200), phospho-c-Jun (1:50; c-Jun (S63/73), and caspase-3 (1:200). After exhaustive rinsing, we incubated the cells with secondary fluorescent antibodies (DyLight 488 and 594 horse anti-rabbit, -goat and -mouse, cat DI 1094, DI 3088, and DI 2488, respectively) at 1:500. Fluorescence analysis was performed on a BD LSRFortessa II flow cytometer (BD Biosciences, Becton, Dickinson and Company, BD Biosciences, 2350 Qume Dr, San Jose, CA 95131-1812, USA). Cells without primary antibodies served as a negative control. For assessment, 10,000 events and quantitative data and figures were obtained using FlowJo 7.6.2 Data Analysis Software (TIBCO® Data Science, Palo Alto, Ca, USA). Events analysis was performed by determining the cell population (Forward Scatter analysis, Y axis) that exceeded the basal fluorescence (488 nm or 594 nm, X axis) of the negative control. Accordingly, contour diagrams were created from event analysis, and the cells located in the box (quadrants labeled as + or (+)) represent the cell population exceeding the basal fluorescence.

### 2.6. Western Blot Analysis

To test the effect of EGCG on Aβ42 oligomerization, 1µg of Aβ42 (0.1 µg/µL) was incubated alone or in combination with 50 µM EGCG or 6E10 antibody (1:10). Then, samples were heated at 95 °C for 5 min in 2 × SDS and 20× reducing agent and loaded on to 12% gels at 120 V for 90 min, bands were transferred onto nitrocellulose membranes (Hybond-ECL, Amersham Biosciences, 800 Centennial Ave, Piscataway, NJ, USA,) at 270 mA for 90 min using an electrophoretic transfer system (BIO-RAD) according to Bio-Rad protocol (http://www.bio-rad.com/webroot/web/pdf/lsr/literature/Bulletin_6376; accessed on 20 August 2021). The membranes were incubated overnight at 4 °C with anti-APP751 primary antibody (1:5000). Secondary infrared antibody (goat anti-mouse IRDye ^®^ 800CW, cat #926-32270; LI-CORBiosciences, 4647 Superior Street, Lincoln, Ne, USA) at 1:10,000 was used for western blotting analysis, and data were acquired using Odyssey software. The assessment was repeated two times in independent experiments. To evaluate the effect of EGCG on the conformation-dependent differences among Aβ assemblies, we prepared a homogenous synthetic unaggregated (i.e., monomers) and large oligomeric Aβ42 assemblies according to ref. [30]. Briefly, after solubilization of the peptide (Sigma Cat #A9810, 3050 Spruce Street, St. Louis, MO 63103, USA) in DMSO, the “unaggregated” peptide was obtained by dissolving the DMSO-solubilized peptide in water and used immediately (0 days). To obtain the “large oligomers”, 10 mM Tris was added to DMSO-solubilized peptide solution and incubated for 15 days at 4 °C. The determination of the aggregation state of Aβ42 in the presence of EGCG or antibody 6E10 Aβ42/APP was performed by Western analysis of SDS-PAGE. The assessment was repeated two times in independent experiments.

### 2.7. Evaluation of Intracellular Hydrogen Peroxide (H_2_O_2_) by Fluorescence Microscopy

To assess the levels of intracellular H_2_O_2_, we used 2′,7′-dichlorofluorescein diacetate (5 μM, DCFH2-DA; Invitrogen, 168 Third Avenue. Waltham, MA, USA) according to ref. [15]. ChLNs were left in RCm for 4 days. Then, the cells (5 × 10^3^) were incubated with the DCFH2-DA reagent for 30 min at 37 °C in the dark. Cells were then washed, and DCF fluorescence intensity was determined by analysis of fluorescence microscopy images. The assessment was repeated three times in independent experiments. The nuclei were stained with 0.5 µM Hoechst 33,342 staining compound. The assessment was repeated three times in independent experiments blind to the experimenter.

### 2.8. Analysis of Mitochondrial Membrane Potential (ΔΨm) by Fluorescence Microscopy

The ChLNs were left in a regular culture medium (RCm) for 4 days. Then, the cells (5 × 10^3^) were incubated with the passively diffusing and active mitochondria-accumulating dye deep red MitoTracker compound (20 nM, final concentration) for 20 min at RT in the dark (Invitrogen, cat # M22426, 168 Third Avenue. Waltham, MA, USA) [15]. Cells were then washed twice with PBS. MitoTracker fluorescence intensity was determined by analysis of fluorescence microscopy images (Floyd Cells Imaging Station microscope, catalogue 4471136, Thermo Fisher Scientific, 168 Third Avenue. Waltham, MA, USA). The assessment was repeated three times in independent experiments. The nuclei were stained with 0.5 µM Hoechst 33,342 staining compound. The assessment was repeated three times in independent experiments blind to the experimenter and flow cytometer analyst.

### 2.9. Measurement of Extracellular (e)Aβ42 Peptide in Culture Medium

The level of (e)Aβ1-42 peptide was measured according to ref. [32] with minor modifications. Briefly, WT and PSEN1 E280A ChLNs were left in RCm for 4 days. Then, 100 µL of conditioned medium was collected, and the levels of secreted Aβ42 peptides were determined by a solid-phase sandwich ELISA (Invitrogen, Cat# KHB3544, 168 Third Avenue. Waltham, MA, USA) following the manufacturer’s instructions. The assessment was repeated four times in independent experiments blind to the experimenter. 

### 2.10. Intracellular Calcium Imaging

Intracellular calcium (Ca^2+^) concentration changes evoked by cholinergic stimulation were assessed according to refs. [33,34], with minor modifications. For the measurement, the fluorescent dye Fluo-3 (Fluo-3 AM; Thermo Fisher Scientific, cat: F1242, 168 Third Avenue. Waltham, MA, USA) was employed. The dye was dissolved in DMSO (1 mM) to form a stock solution. Before the experiments, the stock solution was diluted in neuronal buffer solution (NBS buffer in mM: 137 NaCl, 5 KCl, 2.5 CaCl_2_, 1 MgCl_2_, pH 7.3, and 22 glucose). The working concentration of the dye was 2 μM. The WT and PSEN1 E280A ChLNs were incubated for 30 min at 37 °C with the dye containing NBS and then washed five times. Intracellular Ca^2+^ transients were evoked by acetylcholine (1 mM final concentration) at 4 days post differentiation. The measurements were carried out using the 20× objective of the microscope. Several regions of interest (ROIs) were defined in the visual field of the camera. One of the ROIs was cell free, and the fluorescence intensity measured here was considered background fluorescence (F_bg_). The time dependence of the fluorescence emission was acquired, and the fluorescence intensities (hence the Ca^2+^ levels) were represented by pseudo colors. To calculate the changes of the average Ca^2+^-related fluorescence intensities, the F_bg_ value was determined from the cell-free ROI, and then the resting fluorescence intensities (F_rest_) of the cell-containing ROIs were obtained as the average of the points recorded during a consecutive period of 10 s before the addition of acetylcholine. The peaks of the fluorescence transients were found by calculating the average of six consecutive points and identifying those points that gave the highest average value (Fmax). The amplitudes of the Ca^2+^-related fluorescence transients were expressed relative to the resting fluorescence (ΔF/F) and were calculated by the following formula: ΔF/F = (F_max_ − F_rest_)/(F_rest_ − F_bg_). For the calculation of the fluorescence intensities, ImageJ was used. The terms fluorescence intensity was used as an indirect indicator of intracellular Ca^2+^ concentration. The assessment was repeated three times in independent experiments blind to the experimenter.

### 2.11. Measurement of Interleukin-6 (IL-6) in Culture Medium

The WT and PSEN1 E280A ChLNs were left in MCm for 4 days. Then, the conditioned medium was collected and used to stimulate ALCs for 4 days alone or in combination with 50 μM EGCG. Levels of secreted IL-6 were determined through the ELISA kit (Cat. No. 430504, Biolegend, CA, USA) following the manufacturer’s instructions. The assessment was repeated 3 times in independent experiments blind to the experimenter. Finally, IL-6 levels were normalized to the protein concentration of cells in the culture dish.

### 2.12. Photomicrography and Image Analysis

Light microscopy photographs were taken using a Zeiss Axiostart 50 Fluorescence Microscope equipped with a Canon PowerShot G5 digital camera (Zeiss Wöhlk-Contact-Linsen, Gmb Schcönkirchen, Germany), and fluorescence microscopy photographs were taken using a Zeiss Axiostart 50 Fluorescence Microscope equipped with a Zeiss AxioCam Cm1 and (Zeiss Wöhlk-Contact-Linsfluoreen, Gmb Schcönkirchen, Germany) and Floyd Cells Imaging Station microscope. Fluorescence images were analyzed by ImageJ software (http://imagej.nih.gov/ij/, accessed on 15 September 2021). The figures were transformed into 8-bit images, and the background was subtracted. The cellular measurement regions of interest (ROIs) were drawn around the nucleus (for the case of transcription factors and apoptosis effectors) or overall cells (for cytoplasmic probes), and the fluorescence intensity was subsequently determined by applying the same threshold for cells in the control and treatment conditions. Mean fluorescence intensity (MFI) was obtained by normalizing total fluorescence to the number of nuclei.

### 2.13. Data Analysis

In this experimental design, two vials of MSCs were thawed (PSEN1-WT and -E280A), cultured and the cell suspension was pipetted at a standardized cellular density of 2.6 × 10^4^ cells/cm^2^ into different wells of a 24-well plate. Cells (i.e., the biological and observational unit [35] were randomized to wells by simple randomization (sampling without replacement method), and then wells (i.e., the experimental units) were randomized to treatments by a similar method. Experiments were conducted in triplicate wells. The data from individual replicate wells were averaged to yield a value of n = 1 for that experiment and this was repeated on three occasions blind to the experimenter and/or flow cytometer analyst for a final value of n = 3 [35]. Based on the assumption that the experimental unit (i.e., the well) data comply with the independence of observations, the dependent variable is normally distributed in each treatment group (Shapiro-Wilk test), and variances are homogeneous (Levene’s test), the statistical significance was determined by one-way analysis of variance (ANOVA) followed by Tukey’s post hoc comparison calculated with GraphPad Prism 5.0 software. Differences between groups were only deemed significant when a *p*-value of <0.05 (*), <0.001 (**) and <0.001 (***). All data are illustrated as the mean ± S.D.

## 3. Results

### 3.1. EGCG Scavenges Reactive Oxygen Species (ROS) and Reestablishes the Mitochondrial Membrane Potential (ΔΨm) in PSEN1 E280A ChLNs 

**Figure 1:**
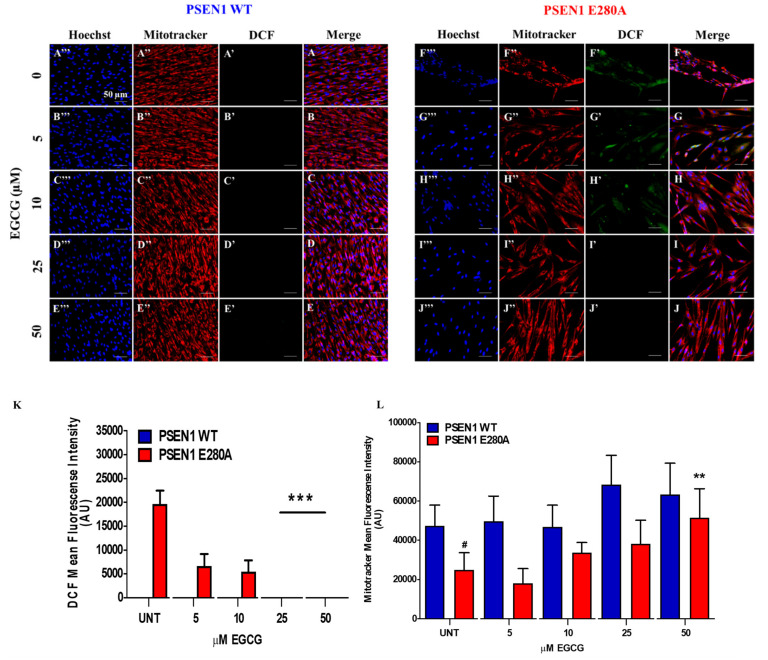
EGCG scavenges reactive oxygen species (ROS) and reestablishes the mitochondrial membrane potential (ΔΨm) in a dependent-concentration manner in PSEN1 E280A ChLNs. After 7 days of transdifferentiation, WT PSEN1 and PSEN1 E280A ChLNs were left untreated or treated with EGCG at increasing concentrations (0, 5, 10, 25, and 50 μM) in a regular culture medium (RCm) for 4 days. Representative DCF+ cells (**A’**–**J’**), MitoTracker (**A”**–**J”**), Hoechst (**A’’’**–**J’’’**), and merged (**A**–**J**) pictures of WT PSEN1 and PSEN1 E280A ChLNs treated as described. (**K**) Quantification of DCF fluorescence intensity. (**L**) Quantification of MitoTracker fluorescence intensity. Data are expressed as the mean ± SD; ^#^ *p* < 0.05; ** *p* < 0.01; *** *p* < 0.001. ^#, ^** represents differences compared to UNT PSEN1 E280A cells. The histograms and figures represent 1 out of 3 independent experiments.

To determine whether EGCG recovers ΔΨm and scavenge ROS, cholinergic cells were exposed to increasing concentrations of EGCG. As shown in Figure 1A–E, EGCG (5–50 μM) neither generated ROS (Figure 1A’–E’,K) nor affected ΔΨm in wild-type ChLNs (Figure 1A”–E”,L). However, the polyphenol treatments dose-dependently reduced the endogenous production of ROS (Figure 1F’–J’,K), and increased the ΔΨm in PSEN1 E280A ChLNs (Figure 1F”–J”,L). We found that 25–50 μM, but not low concentrations (5, 10 μM), were the maximal optimal concentrations to completely blunt ROS yield and to raise ΔΨm in mutant cells (Figure 1F–L). Therefore, we selected EGCG (50 μM) concentration for further experiments.

### 3.2. EGCG Partially Reduces Intracellular sAPPβf Aggregation but Completely Inhibits Oxidized DJ-1 in PSEN1 E280A ChLNs

**Figure 2:**
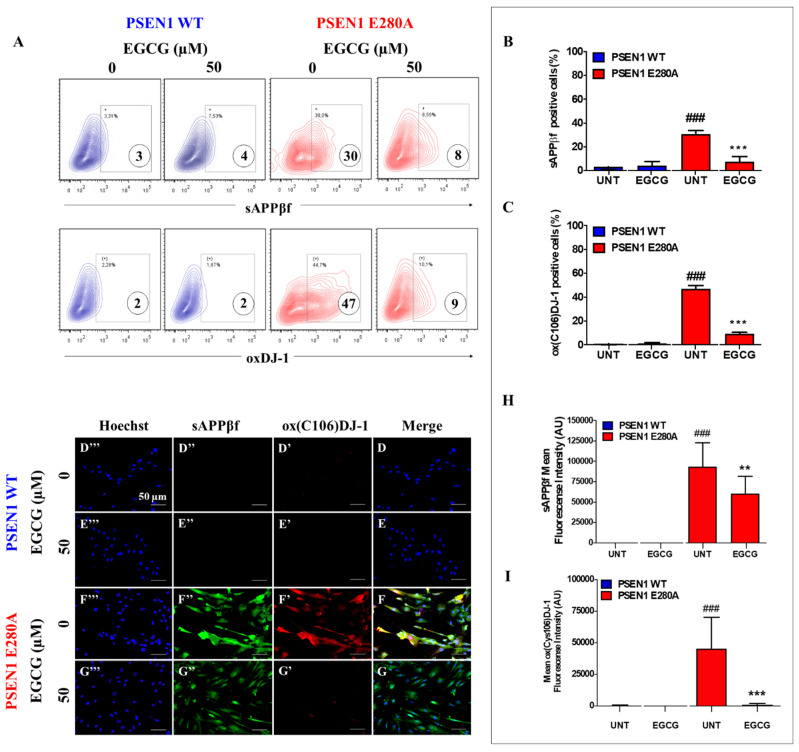
EGCG partially reduces the intracellular sAPPβf aggregation but completely inhibits oxidized DJ-1 in PSEN1 E280A ChLNs. After 7 days of transdifferentiation, WT PSEN1 and PSEN1 E280A ChLNs were left untreated or treated with EGCG in RCm for 4 days. Then, the cells were labeled with primary antibodies against Aβ42 and oxDJ-1Cys106, and fluorescent secondary antibodies. The fluorescent contour plot shown in (**A**) was quantified (**B**,**C**). Additionally, cells were double-stained as indicated in the figure (**D**–**G**) with primary antibodies against oxDJ-1Cys106 (red; **D’**–**G’**) and APP751/Aβ42 (green; **D’’**–**G’’**). The nuclei were stained with Hoechst 33,342 (blue; **D’’’**–**G’’’**). (**H**) Quantification of Aβ42 fluorescence intensity. (**I**) Quantification of oxDJ-1Cys106 fluorescence intensity. Data are expressed as the mean ± SD; ** *p* < 0.01; ^###, ^*** *p* < 0.001. ^###^ represents differences compared to UNT PSEN1 WT cells; *** represents differences compared to UNT PSEN1 E280A cells. The figures represent 1 out of 3 independent experiments.

Next, we assessed whether EGCG inhibits the intracellular aggregation of sAPPβf and avoids oxidation of the stress sensor protein DJ-1. To this aim, WT and mutant ChLNs were left untreated or exposed to EGCG. Flow cytometry (FC) analysis reveals that EGCG significantly reduced the aggregation of (i)sAPPβf (Figure 2A,B) and almost completely blunted the oxidation of DJ-1 in mutant ChLNs. Neither (i)sAPPβf nor oxidized DJ-1 were detected in those WT ChLNs (Figure 2A,C). These results were confirmed by fluorescent microscopy (FM, Figure 2D–I).

### 3.3. EGCG Blocks Apoptosis Signaling in PSEN1 E280A ChLNs

**Figure 3:**
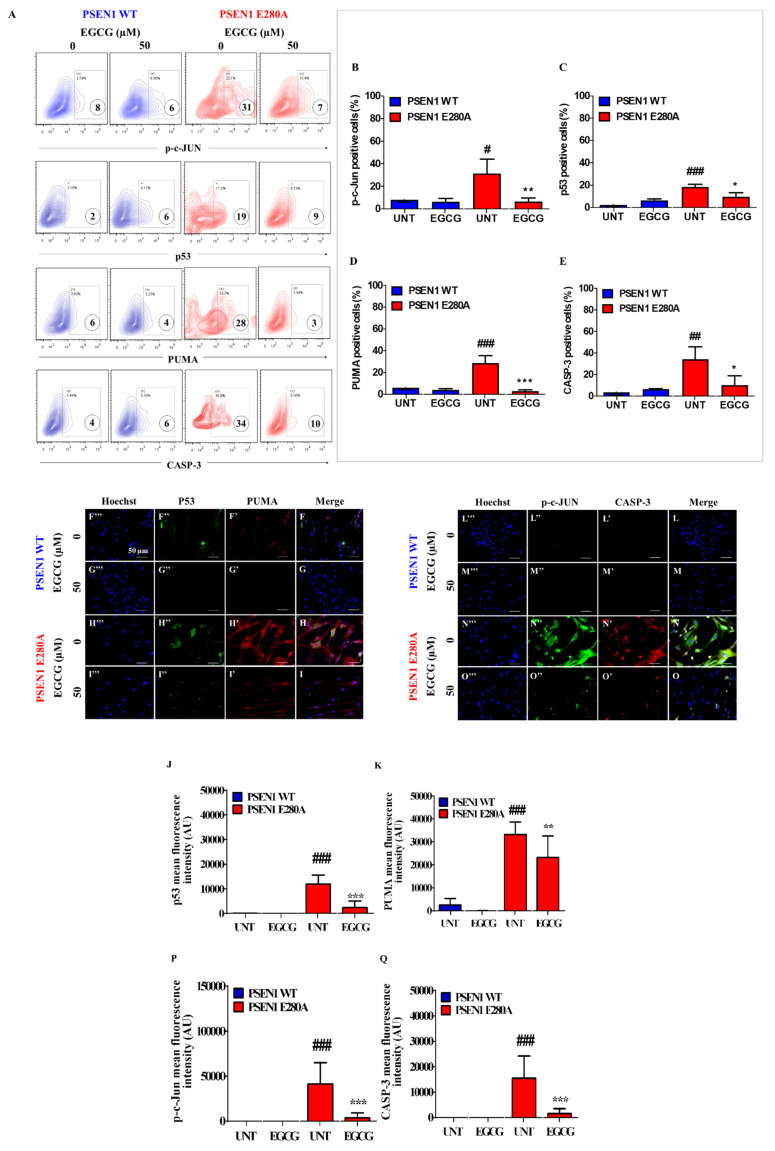
EGCG reduced the activation of P53, PUMA, c-JUN, and CASPASE-3 in PSEN1 E280A ChLNs. After 7 days of transdifferentiation, WT PSEN1 and PSEN1 E280A ChLNs were left untreated or treated with EGCG in a regular culture medium for 4 days. Then, cells were labeled with primary antibodies against c-JUN/total c-JUN, P53, PUMA, CASPASE-3 (CASP-3), and fluorescent secondary antibodies. The fluorescent contour plot shown in (**A**) were quantified (**B**–**E**). Additionally, cells were double-stained as indicated in the figure (**F**–**O**) with primary antibodies against P53 (green; **F”**–**I”**), PUMA (red; **F’**–**I’**), c-JUN (green; **L”**–**O”**), and CASP-3 (red; **N’**–**O’**). The nuclei were stained with Hoechst 33,342 (blue; **F’’’**–**O’’’**). (**J**,**K**,**P**,**Q**) Quantification of P53 (**J**), PUMA (**K**), c-JUN (**P**), and CASP-3 (**Q**) fluorescence intensity. Data are expressed as the mean ± SD; ^#,^* *p* < 0.05; ^##, ^** *p* < 0.01; ^###, ^*** *p* < 0.001; ^###^ represents differences compared to UNT PSEN1 WT cells; **^,^*** represents differences compared to UNT PSEN1 E280A cells. The figures represent 1 out of 3 independent experiments. Image magnification, 200×.

Apoptosis is an important feature in mutant ChLNs [15]. To investigate whether EGCG regulates apoptosis in mutant cells, we used the activation of the transcription factors P53 and c-JUN, pro-apoptotic BH3-only protein PUMA, and protease CASPASE-3 as cell death markers. Flow cytometry (FC) analysis reveals that PSEN1 E280A ChLNs displayed high levels of protein c-JUN (Figure 3A,B), P53 (Figure 3A,C), PUMA (Figure 3A,D) and CASP-3 (Figure 3A,E), but not in WT ChLNs (Figure 3A–E). However, EGCG significantly reduced c-JUN (Figure 3A,B), P53 (Figure 3A,C), PUMA (Figure 3A,D), and CASP-3 (Figure 3A,E) in PSEN1 E280A neurons compared to treated WT ChLNs cells. These observations were confirmed by fluorescent microscopy (Figure 3F–Q). 

### 3.4. EGCG Inhibits TAU Phosphorylation in PSEN1 E280A ChLNs 

**Figure 4:**
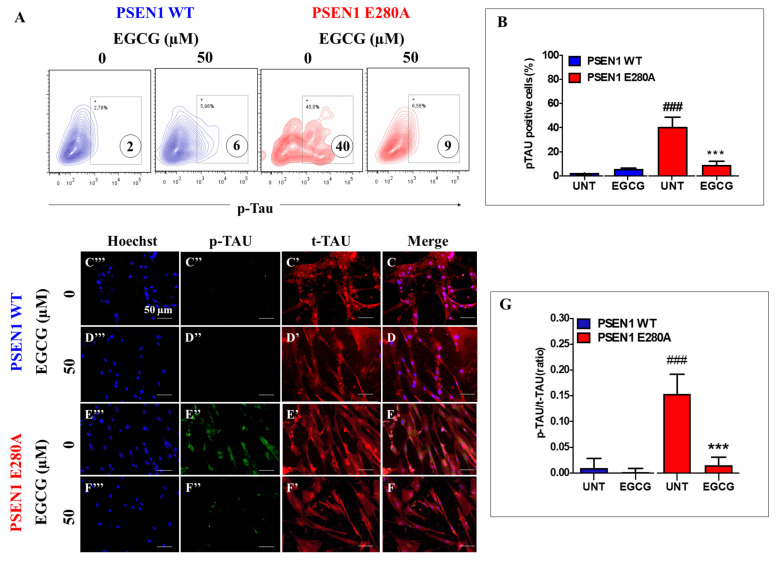
EGCG reduces the phosphorylation of TAU protein in PSEN1 E280A ChLNs. After 7 days of transdifferentiation, WT PSEN1 and PSEN1 E280A ChLNs were left untreated or treated with EGCG in a regular culture medium for 4 days. Then, the cells were labeled with primary antibodies against phosphorylated TAU (p-TAU), and fluorescent secondary antibodies. The fluorescent contour plot shown in (**A**) were quantified (**B**). Additionally, cells were double-stained as indicated in the figure (**C**–**F**) with primary antibodies against p-TAU (green; **C’**–**F’**) and t-TAU (red; **C’’**–**F’’**). The nuclei were stained with Hoechst 33,342 (blue; **C’’’**–**F’’’**). (**G**) Quantification of the p-TAU/t-TAU fluorescence ratio. Data are expressed as the mean ± SD; ^###, ^*** *p* < 0.001; ^###^ represents differences compared to UNT PSEN1 WT cells; *** represents differences compared to UNT PSEN1 E280A cells. The figures represent 1 out of 3 independent experiments.

Previously, it was shown that (i)sAPPβf induces the phosphorylation of TAU [15]. To investigate whether EGCG inhibits TAU phosphorylation, WT and PSEN1 E280A ChLNs were left untreated or treated with the polyphenol. As shown in Figure 4, while the level of TAU phosphorylation was detected to a similar extent in WT ChLNs under EGCG exposure (Figure 4A,B), EGCG dramatically reduced phosphorylation of protein TAU in mutant ChLNs (Figure 4A,B) compared to untreated or treated WT neurons. These observations were confirmed by fluorescent microscopy (Figure 4C–G). 

### 3.5. EGCG Does Not Reduce the Levels of Extracellular Aβ42 (eAβ42) Protein Fragment in PSEN 1 E280A ChLNs

**Figure 5:**
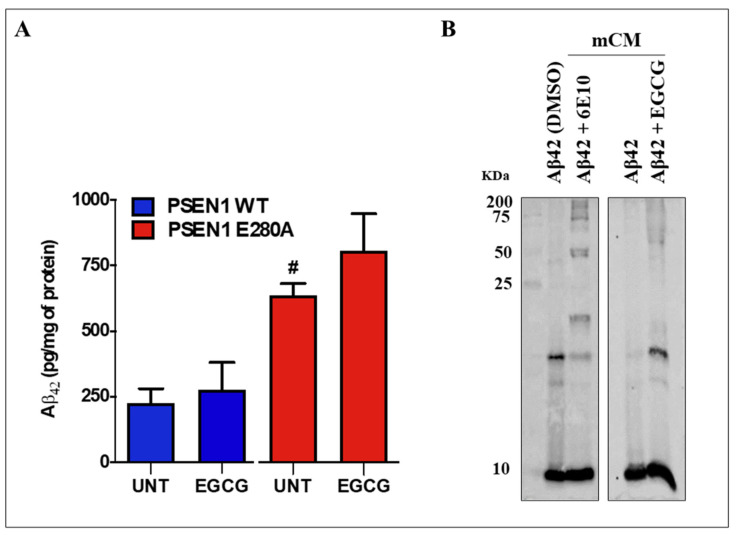
EGCG does not reduce the levels of extracellular Aβ42 peptides in PSEN1 E280A ChLNs. After 7 days of transdifferentiation, WT PSEN1 and PSEN1 E280A ChLNs were left untreated or treated with EGCG in RCm for 4 days. (**A**) ELISA quantification of extracellular Aβ42 peptides in supernatants. (**B**) Western blot showing the Aβ42 migration profile alone or after incubation with EGCG or 6E10 antibody. Data are presented as means ± SD. ^#^ *p* < 0.05, ^#^ represents differences compared to UNT PSEN1 WT cells. The histograms represent 1 out of 4 independent experiments, and blot represents 1 out of 2 independent experiments.

We further evaluated whether EGCG affects the amounts of secreted Aβ42 by nerve cells [15]. As shown in Figure 5, EGCG did not reduce the amounts of (e)Aβ42 secreted by mutant cells (870 ± 234 pg eAβ42/mg protein) compared to untreated mutant neurons (635 ± 46 pg eAβ42/mg protein) according to the ELISA technique (Figure 5A). Similar amounts of secreted eAβ42 were detected in untreated (225 ± 55 pg eAβ42/mg protein) as well as EGCG treated (274 ± 80 pg eAβ42/mg protein) WT ChLNs (*p* < 0.01, Figure 5A). This observation prompted us to inquire further on the interactions of EGCG and (e)Aβ42. Thus, synthetic Aβ42 fragment was either incubated with DMSO or in a minimal culture medium (mCM) in the presence of antibody Aβ42 6E10 or EGCG for 24 h. As shown in Figure 5B, Western blot (WB) analysis reveals that Aβ42 in DMSO forms aggregates of circa 8–25 kDa, whereas in mCM Aβ42 forms mostly aggregate of approximately 8 kDa (probably Aβ42 dimers). Interestingly, Aβ42 incubated with antibody Aβ42 6E10 or EGCG in mCM not only showed oligomerization of Aβ42 (e.g., 8 kDa) but also high molecular weight Aβ42 fragments (circa 50–200 kDa).

### 3.6. EGCG Recovers Ca^2+^ Dysregulation in PSEN1 E280A ChLNs 

**Figure 6:**
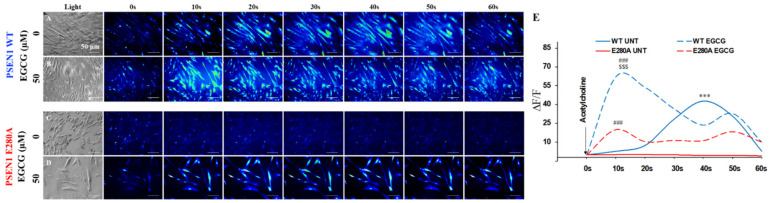
EGCG recovers Ca^2+^ dysregulation in PSEN1 E280A ChLNs. After 7 days of transdifferentiation, WT PSEN1 and PSEN1 E280A ChLNs were left untreated or treated with EGCG in a regular culture medium for 4 days. (**A**–**D**) Time-lapse images (0, 10, 20, 30, 40, 50, and 60 s) of Ca^2+^ fluorescence in WT PSEN1 and PSEN1 E280A ChLNs on day 4 in response to ACh treatment. ACh was puffed into the culture at 0 s (arrow). Then, the Ca^2+^ fluorescence of the cells was monitored at the indicated times. Color contrast indicates fluorescence intensity: dark blue < light blue < green < yellow < red. (**E**) Normalized mean fluorescence signal (∆F/F) over time from the cells indicating temporal cytoplasmic Ca^2+^ elevation in response to ACh treatment. Data are presented as the mean ± SD. ***^, ###, $$$^ *p* < 0.001; *** represents differences compared to UNT PSEN1 E280A cells; ^###^ represents differences compared to UNT PSEN1 WT or E280A cells; ^$$$^ represents differences compared to EGCG-treated PSEN1 E280A cells. The figures represent 1 out of 3 independent experiments.

We then test whether EGCG ameliorates the physiological response of mutant ChLNs to ACh neurotransmitter stimuli. As illustrated in Figure 6, ACh induces a transient elevation of intracellular Ca^2+^ in WT ChLNs (control, the average maximum fluorescence change (ΔF/F) was 43 ± 13 at 40s (n = 20 ChLN cells imaged, N = 3 dishes were used as a standard condition in this and following experiments)) according to cytoplasmic Ca^2+^ responses to Fluo-3-mediated imaging (Figure 6A,E). In contrast, under similar experimental conditions, PSEN1 E280A ChLNs were almost unresponsive to ACh stimuli (ΔF/F= −0.5 ± −0.5 at 40s, Figure 6C,E). However, when WT and mutant neurons were exposed to ACh and EGCG, both WT and mutant ChLNs increased intracellular Ca^2+^, albeit with dissimilar strength. Indeed, while WT ChLNs increases almost 22-fold Ca^2+^ influx (Figure 6B,E, ΔF/F = 62.1 ± 13.4 at 10 s) compared to untreated WT (Figure 6B,E, ΔF/F = 2.8 ± 0.2), mutant ChLNs increases almost 100-fold Ca^2+^ influx (Figure 6D,E, ΔF/F = 20.1 ± 7.0 at 10 s) compared to untreated mutant neurons (Figure 6C,E, ΔF/F = 0.20 ± 0.10 at 10 s). Interestingly, EGCG increases almost 3-fold the intracellular Ca^2+^ influx in WT ChLNs compared to mutant neurons treated at 10 s. 

### 3.7. EGCG Dramatically Reduces Reactive Astrocyte-like Cells Derived from Menstrual Stromal Cells (MenSCs) Exposed to ChLNs’ Supernatant Culture Medium 

**Figure 7:**
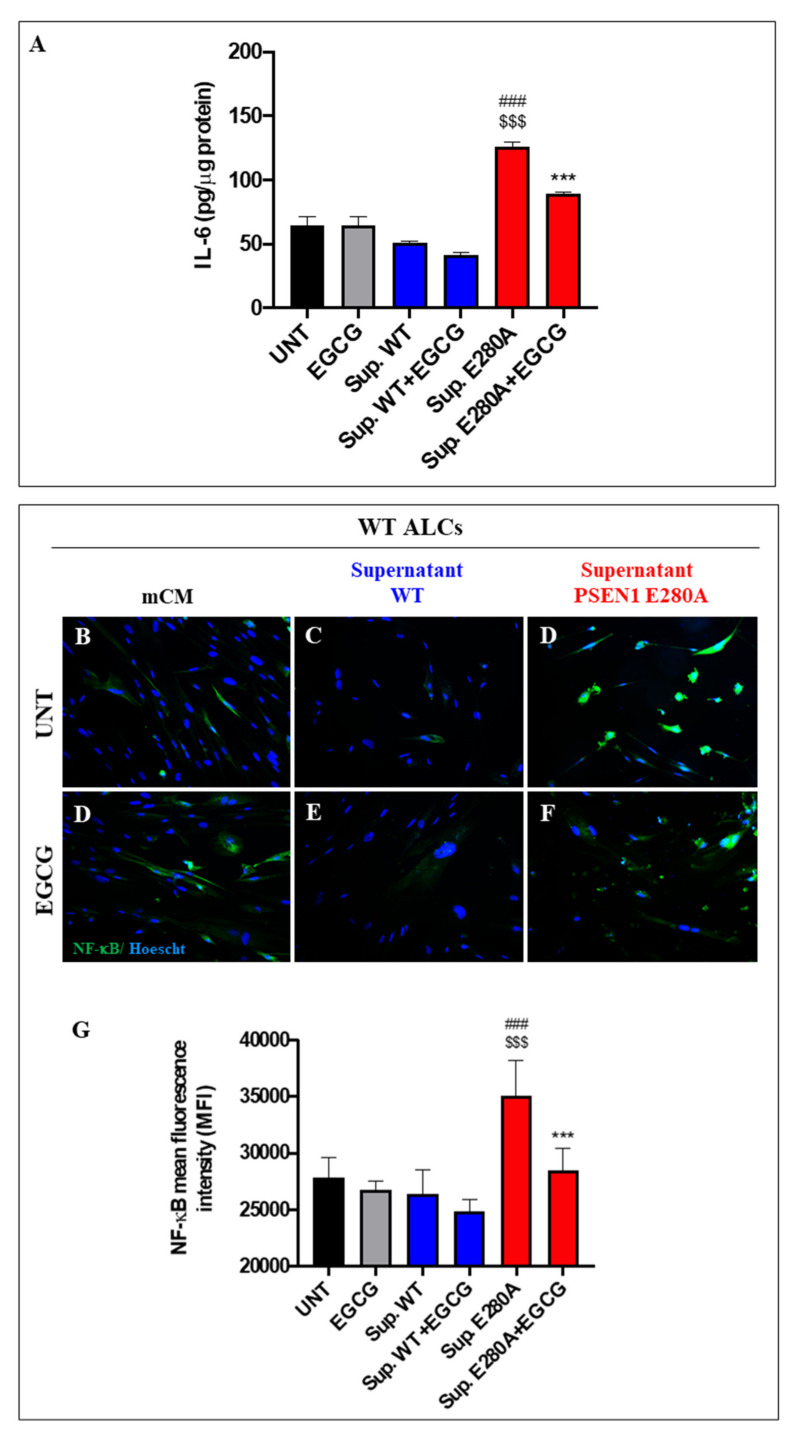
The PSEN1 E280A culture supernatant induces secretion of a proinflammatory molecule (IL-6), and activation of NF-κB in astrocyte-like cells (ALCs). (**A**) After 7 days of transdifferentiation of MenSCs, ALCs were exposed to either mCM, WT supernatant, or PSEN1 E280A culture supernatant for 4 days in the absence (untreated, UNT) or presence of EGCG. Then, supernatants were collected and used to determine IL-6 levels according to the described method. The levels of IL-6 were normalized to the cell protein concentration. (**B**) After 7 days of transdifferentiation of MenSCs, ALCs were exposed to either mCM, WT supernatant, or PSEN1 E280A culture supernatant for 4 days in the absence (untreated, UNT) or presence of EGCG. Then, cells were labeled with primary antibodies against NF-κB, and fluorescent secondary antibodies. The nuclei were stained with Hoechst 33342. Representative merged images of ALCs exposed to mCM (**B**,**D**), WT supernatant (**C**,**E**), and PSEN1 E280A culture supernatant (**D**,**E**) without (**B**–**D**) or with EGCG (**D**–**F**). (**G**) Quantification of NF-κB fluorescence intensity. Data are expressed as the mean ± SD; ^###,^ ***^, $$$^ *p* < 0.001; *** represents differences compared to Sup. E280A; ^###^ represents differences compared to UNT ALCs; ^$$$^ represents differences compared to Sup. WT. The histogram and figures represent 1 out of 3 independent experiments. Image magnification, 200×.

Lastly, we evaluated the anti-inflammatory effect of EGCG on astrocyte-like cells (ALCs) [27]. ALCs were incubated for 4 days with the supernatant culture medium from (7 days) cultured WT and PSEN1 E280A ChLNs (which contains significantly high amounts of Aβ42 (Figure 5A)) in the absence or presence of EGCG. Since IL-6 is a pleiotropic pro-inflammatory cytokine important for the transition from the acute phase to the chronic phase of inflammation in AD [26], we selected IL-6 as a model cytokine in our in vitro neuroinflammation test. Therefore, the amounts of secreted pro-inflammatory cytokine IL-6 by ALCs were evaluated by ELISA technique, and activation of NF-κB (p65), which is a critical transcription factor in immunomodulation, was assessed by immunofluorescent microscopy (IMF), an indicator of neuroinflammation and reactive astrogliosis, respectively [36]. As shown in Figure 7, both mCM and supernatant WT induced neither secretion of pro-inflammatory molecule IL-6 (Figure 7A) nor activation of NF-κB in WT ALCs in presence or absence of EGCG (Figure 7B–D,G). When ALCs were incubated with the PSEN1 E280A ChLNs supernatant only, the amounts of IL-6 significantly increased (Figure 7A) compared to untreated or treated ALCs with WT supernatant. Likewise, PSEN1 E280A ChLNs supernatant induced the activation of NF-κB in ALCs (Figure 7D,G) compared to untreated ALCs (Figure 7C,G). However, EGCG significantly diminished the secretion of IL-6 (Figure 7A) and inactivated NF-κB in ALCs cultured in PSEN1 E280A ChLNs supernatant (Figure 7F,G) to a similar extend as ALCs incubated with EGCG only (Figure 7E,G). 

## 4. Discussion

We report for the first time that the catechin EGCG protects PSEN 1 E280A ChLNs against (i)sAPPβf and eAβ42-induced cytotoxic effects. Indeed, EGCG inhibited the aggregation of (i)sAPPβf, blocked p-TAU, increased ∆Ψm, decreased oxidation of DJ-1Cys106-SH residue, and inhibited the activation of transcription factor c-JUN and P53, blocked BH3-only protein PUMA, and executer protein CASPASE-3. Furthermore, it reversed Ca^2+^ influx dysregulation as a response to ACh stimuli in mutant ChLNs, inhibited pro-inflammatory IL-6, and turned down NF-κB in WT ALCs exposed to mutant ChLNs culture supernatant. Taken together these results suggest that EGCG is a multitarget compound working as an anti-amyloidogenic, anti-apoptosis, anti-phospho-TAU, anti-oxidant agent, ∆Ψm enhancer, transient intracellular Ca^2+^ influx regulator in PSEN 1 E280A ChLNs, and anti-neuroinflammatory agent in ALCs. 

We found for the first time that EGCG inhibited aggregation of (i)sAPPβf (e.g., APP714, APP733, APP751, APP752), thereby reducing its toxicity in mutant ChLNs [15]. Although no information is yet available to explain its mechanism of action on (i)sAPPβf, several data suggest that EGCG reduced the toxicity of amyloidogenic polypeptides by directly binding with unfolded protein and inhibiting the formation of β-sheet structure, an early event of amyloid formation cascade. At the molecular level, auto-oxidized EGCG reacts with free primary amine groups of proteins, forms a Schiff base, and induces fibril remodeling [37,38]. Interestingly, this mechanism not only might be operative with Aβ and TAU [39] in AD but also to other neurodegenerative disorders such as α-synuclein (in Parkinson’s disease), Huntingtin protein (in Huntington’s disease), and islet amyloid polypeptide (IAPP, amylin) in the pancreas most probably through a common mechanism, in which EGCG binds to cross-beta sheets amyloid aggregation intermediates remodeling the oligomeric amyloid or pre-formed amyloid fibrils into non-amyloidogenic species [21,40]. According to this view, we found that EGCG significantly reduces (i)sAPPβf aggregates in PSEN 1 E280A ChLNs demonstrated by FM staining, and FC analysis. However, further investigation is needed to confirm this assumption. Specifically, mounting evidence has shown that EGCG inhibits the oligomerization of Aβ42 [41,42,43]. Consistent with previous work [21], we found that EGCG induces highly stable aggregates of synthetic Aβ42. Interestingly, previous studies have reported that EGCG attenuated Aβ generation by suppressing the transcription and translation of BACE1 [41,44]. However, the present study found that EGCG did not affect the amount of secreted (e)Aβ42. This observation implies that EGCG does not interfere with or regulate the metabolism of PPA in mutant ChLNs. Taken together, these observations suggest that EGCG shifts/stabilizes the conformational structure of (i)sAPPβf and inhibits the cytotoxicity of secreted (e)Aβ42 into non-toxic beta fragments but does not alter intracellular APP metabolism in PSEN 1 E280A ChLNs. Remarkably, PSEN1 E280A displayed hyperphosphorylation of protein TAU [15]. In agreement with Guéroux and co-workers [45], we found that EGCG prevented TAU phosphorylation. Therefore, it is concluded that EGCG can effectively reverse the typical neuropathological toxic amyloidogenic polypeptides (i.e., Aβ, TAU [39]) into non-toxic and non-amyloidogenic species in PSEN1 E280A ChLNs. 

Previously, it has been shown that the earliest sign of OS in PSEN1 E280A ChLNs was the generation of ROS, and the oxidation of stress sensor protein DJ-1 Cys106-SH into DJ-1 Cys106-SO3 by H2O2 [15]. In agreement with He and co-workers [46], who observed that EGCG treatment effectively protected the mouse hippocampal neuronal cell line HT22 against H2O2-induced cell viability by decreasing and attenuating ROS generation, we found that this polyphenol completely removed ROS production and avoided the oxidation of protein DJ-1 in mutant ChLNs. Structure–function studies of EGCG concerning its molecular mechanisms of action have suggested that either its galloyl D-ring [46,47] or the B-ring of EGCG [48] might be the primary site for the free radical scavenging activity of antioxidant reactions. Since EGCG has been shown to inhibit catalase [49], our observations suggest that EGCG might operate as a free radical quencher rather than a catalase-like catalysator in PSEN1 E280A ChLNs. 

Mitochondria are critical organelles in cellular life and death decisions, being both processes susceptible to be modulated by the polyphenol EGCG [50]. Indeed, it has been shown that EGCG protected human lens epithelial HLEB-3 cells [51] and pheochromocytoma PC12 cells [52,53] against H2O2-induced apoptosis mainly through inhibition of CASP-3. In line with these observations, we found that EGCG not only reestablished the ∆Ψm but also dramatically decreased the activity of CASP-3, both features affected/activated in PSEN1 E280A ChLNs [15]. Furthermore, we report for the first time that EGCG blocks the activation of the pro-apoptotic proteins c-JUN, P53, and PUMA [54]; all indirect/direct players in the mitochondria depolarization, and subsequent releasing of apoptogenic proteins (e.g., cytochrome c) which ended up in the activation of CASP-3 in mutant neurons. Taken together, these observations suggest that EGCG protects PSEN1 E280A against endogenously generated OS-induced intrinsic apoptosis [55]. 

A previous study has shown that PSEN1 E280A ChLNs displayed reduced functional response to ACh [15]. Here, we confirm that mutant ChLNs were unresponsive to ACh. Therefore, PSEN1 E280A ChLNs essentially present a dysfunctional intracellular Ca^2+^ influx in response to ACh compared to WT ChLNs most probably by the specific interaction between (e)Aβ42 and nAChRs [56]. Accordingly, we found that PSEN1 E280A ChLNs secreted high amounts of (e)Aβ42 (e.g., ~2.6-f.i.) compared to WT PSEN1 ChLNs. However, EGCG moderately recovered the response of PSEN1 E280A to ACh. Based on the observation that EGCG stabilized and redirected the toxic oligomers of synthetic Aβ42 into non-toxic ones (Figure 5B), we speculate that EGCG either blocks (e)Aβ42-nAChRs or EGCG stabilize (e)Aβ42 to a non-amyloidogenic peptide. Our observations favor the former assumption. However, further investigation is needed to clarify this issue. Whatever the mechanism, we demonstrate for the first time that EGCG recovers Ca^2+^ influx in PSEN1 E280A ChLNs in a short window of time (e.g., 10 s) after being puffed ACh. Outstandingly, EGCG was much more effective in recovering mutant ChLNs (100-fold increase Ca^2+^ influx) than enhancing the response of WT ChLNs (22-fold increase Ca^2+^ influx) to ACh. Nonetheless, EGCG increased almost 3-fold the intracellular Ca^2+^ influx in WT compared to mutant neurons. Taken together our observations suggest that EGCG protects PSEN1 E280A against (e)Aβ42-induced dysfunctional Ca^2+^ influx with no involvement in the APP metabolism. EGCG did not alter the secretion of Aβ42 in untreated or treated mutant ChLNs. Therefore, EGCG might be an effective agent to protect neuronal and/or synaptic function. 

Using ALCs as a cellular model of human brain astrocytes [29], we demonstrate for the first time that the supernatant culture medium from PSEN1 E280A ChLNs, but not the supernatant culture medium from WT ChLNs, induces the activation of NF-κB—a proinflammatory transcription factor [57] and the secretion of pro-inflammatory cytokine IL-6 [26] in WT MenSCs-derived astrocyte cells. Here, we also report for the first time that EGCG—a well-established anti-inflammatory molecule (e.g., [22]) dramatically reduced the activation of NF-κB and the secretion of pro-inflammatory cytokine in ALCs. Our observations comply with the notion that EGCG attenuates (e)Aβ42-induced neuroinflammation and neurotoxicity in PSEN1 E280A ChLNs. 

## 5. Conclusions

**Figure 8:**
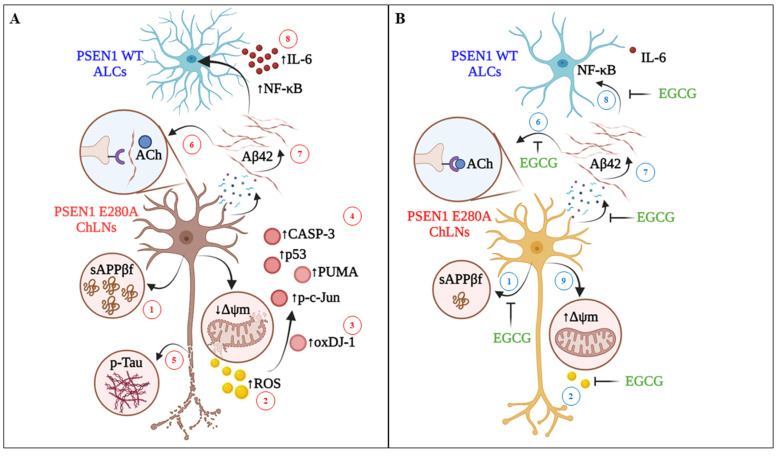
Schematic representation of the protective effect of EGCG on PSEN 1 E280A ChLNs. (**A**) Intracellularly accumulation of sAPPβf (step 1) generates H2O2 (s2), which in turn oxidizes the OS sensor protein DJ-1 at Cys106-SH residue into DJ-1Cys106-SO3 (s3) and activates a domino-like, pro-death signaling mechanism (s4) by triggering the activation of transcription factor P53 and c-JUN, BH-3-only protein PUMA and CASP-3. Interestingly, (i)sAPPβf induces the phosphorylation of protein TAU (p-TAU, s5). Besides, PSEN 1 E280A ChLNs do not respond to ACh stimuli i.e., intracellular transient Ca^2+^ increase is missing due to extracellular interaction between (e)Aβ42 and nicotinic (n)ACh receptors (s6). Therefore, the (i) sAPPβf/Aβ42-induced signaling process (s1–s6) leads ChLNs to structural alterations, cell death (apoptosis), as well as intracellular Ca^2+^ dysfunction. To aggravate matters, the (e)Aβ42 (s7) induces ALCs to secrete pro-inflammatory cytokine IL-6 through activation of transcription factor NF-κB (s8). (**B**) Upon exposure to EGCG, mutant ChLNs show normal features such as no oxidized protein DJ-1 (s3), unaltered ∆Ψm (s9), and intact nuclei morphology. Moreover, EGCG partially inhibits the aggregation of (i)sAPPβf (s1) and blocks the generation of H2O2 (s2). As a result, there is no further activation of pro-death proteins (s4), and phosphorylation of protein TAU (s5). Furthermore, EGCG reestablishes the PSEN1 E280A ChLNs response to ACh (s6–s7) and protects ALCs against (e)Aβ42-induced pro-inflammation stimuli (s8).

In this investigation we have reproduced not only the neuropathological hallmarks of FAD (Figure 8A), but have also shown that EGCG protects ChLNs PSEN1 E280A -a cellular model of FAD by blocking, at least, seven pathological targets: (i)sAPPβf, eAβ42, oxDJ-1, p-TAU, down ∆Ψm, Ca^2+^ dysregulation, and reactive ALCs (Figure 8B). Remarkably, all these beneficial effects of EGCG have been extensively reported in vivo models (e.g., [58]). Therefore, our in vitro test is a valid subrogate cellular model for searching potential anti-FAD drugs. Interestingly, toxicological and human safety data for tea preparations ingested as a solid bolus dose has shown a safe intake level of 338 mg EGCG/day for adults based on human adverse event (AE) data, and observed safe level (OSL) of 704 mg EGCG/day might be considered for tea preparations in beverages [59]. A key question then arises: can EGCG be translated to the clinic in FAD PSEN1 E280A patients [60]? Our present results and previous experimental approaches with EGCG loaded in nanoparticles (e.g., [61,62,63]) encourage its use in the treatment of FAD patients. Although the EGCG dose levels and administration route remain to be established, the EGCG nonetheless might be a promising therapeutic candidate for FAD.
